# Supplementary material for: A Humanized Yeast Phenomic Model of Deoxycytidine Kinase to Predict Genetic Buffering of Nucleoside Analog Cytotoxicity
Source: Genes (Basel). 2019 Sep 30;10(10):770. doi: 10.3390/genes10100770 (PMC6826991; doi:10.3390/genes10100770)
Supplement: Supplementary file 1 [file genes-10-00770-s001.zip › Additional_Files/Additional_File7_GOTermHeatmaps.zip!/Additional_File7_GOTermHeatmaps/transcription_cofactor_activity.pdf]

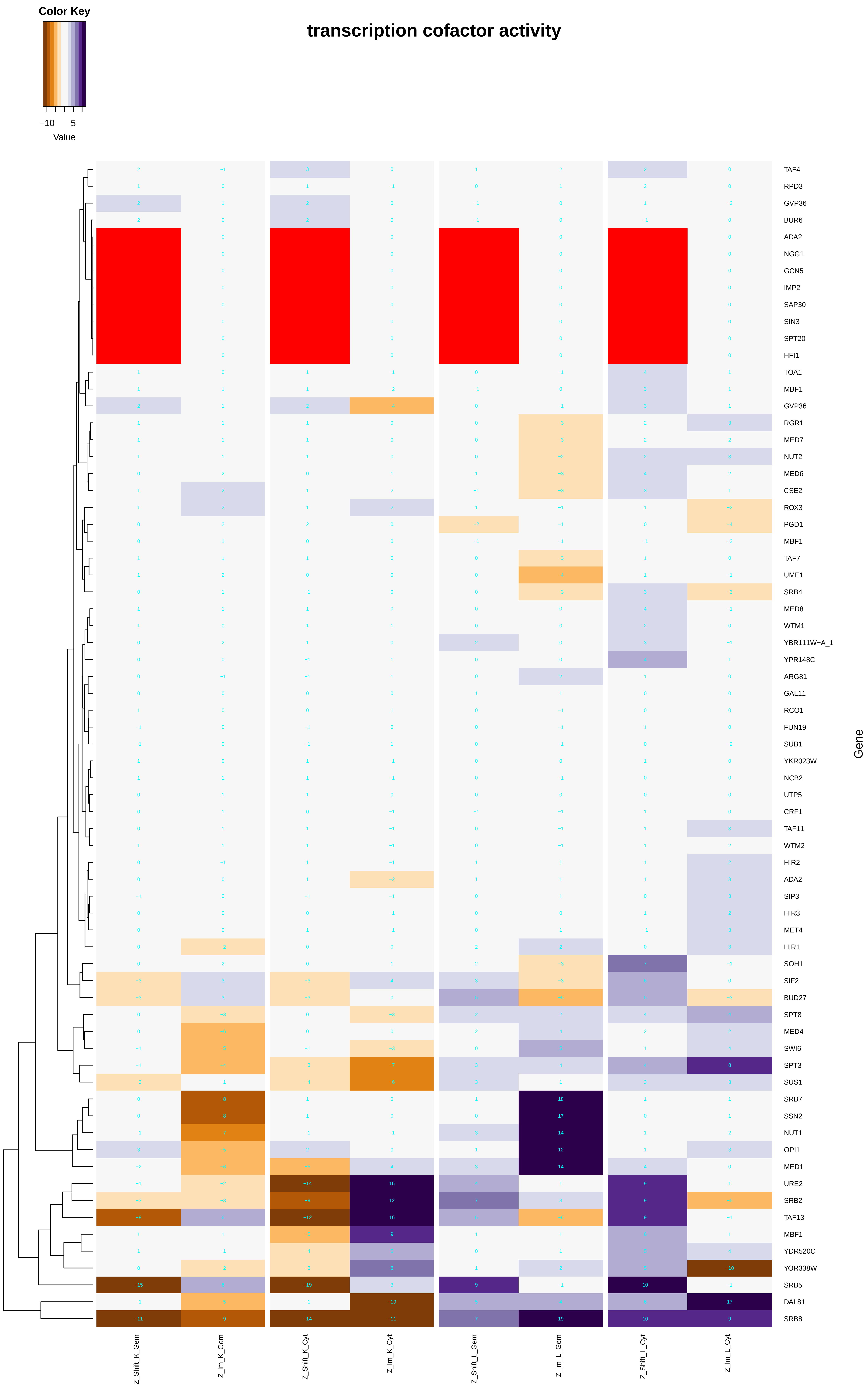
transcription cofactor activity
Color Key
−10
5
Value
TAF4
RPD3
GVP36
BUR6
ADA2
NGG1
GCN5
IMP2'
SAP30
SIN3
SPT20
HFI1
TOA1
MBF1
GVP36
RGR1
MED7
NUT2
MED6
CSE2
ROX3
PGD1
MBF1
TAF7
UME1
SRB4
MED8
WTM1
YBR111W−A_1
YPR148C
ARG81
GAL11
RCO1
FUN19
SUB1
YKR023W
NCB2
UTP5
CRF1
TAF11
WTM2
HIR2
ADA2
SIP3
HIR3
MET4
HIR1
SOH1
SIF2
BUD27
SPT8
MED4
SWI6
SPT3
SUS1
SRB7
SSN2
NUT1
OPI1
MED1
URE2
SRB2
TAF13
MBF1
YDR520C
YOR338W
SRB5
DAL81
SRB8
Gene
Z_Shift_K_Gem
Z_Im_K_Gem
Z_Shift_K_Cyt
Z_Im_K_Cyt
Z_Shift_L_Gem
Z_Im_L_Gem
Z_Shift_L_Cyt
Z_Im_L_Cyt

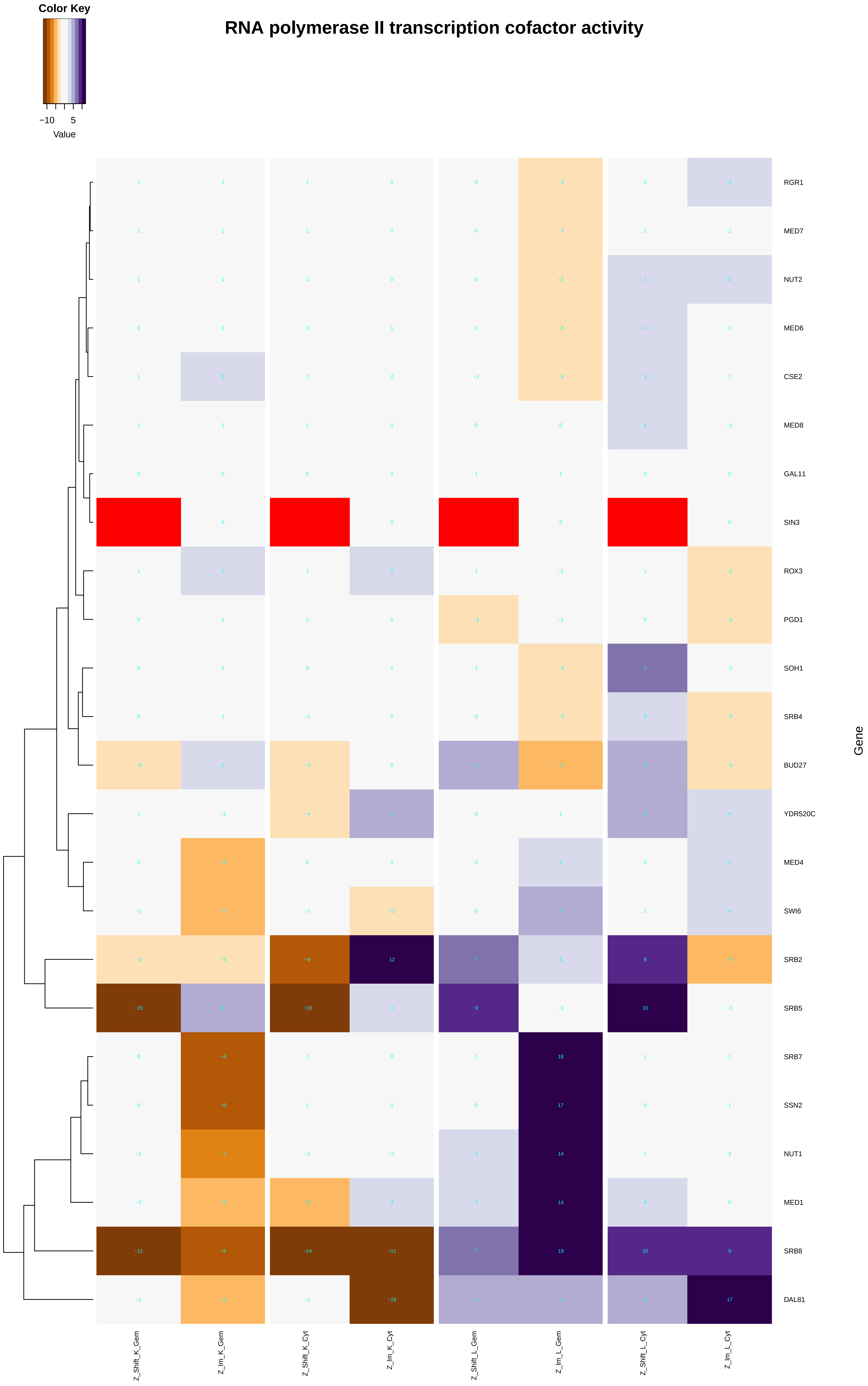

Color Key
−10
5
Value
RNA polymerase II transcription cofactor activity
RGR1
MED7
NUT2
MED6
CSE2
MED8
GAL11
SIN3
ROX3
PGD1
SOH1
SRB4
BUD27
YDR520C
MED4
SWI6
SRB2
SRB5
SRB7
SSN2
NUT1
MED1
SRB8
DAL81
Gene
Z_Shift_K_Gem
Z_Im_K_Gem
Z_Shift_K_Cyt
Z_Im_K_Cyt
Z_Shift_L_Gem
Z_Im_L_Gem
Z_Shift_L_Cyt
Z_Im_L_Cyt

transcription coactivator activity

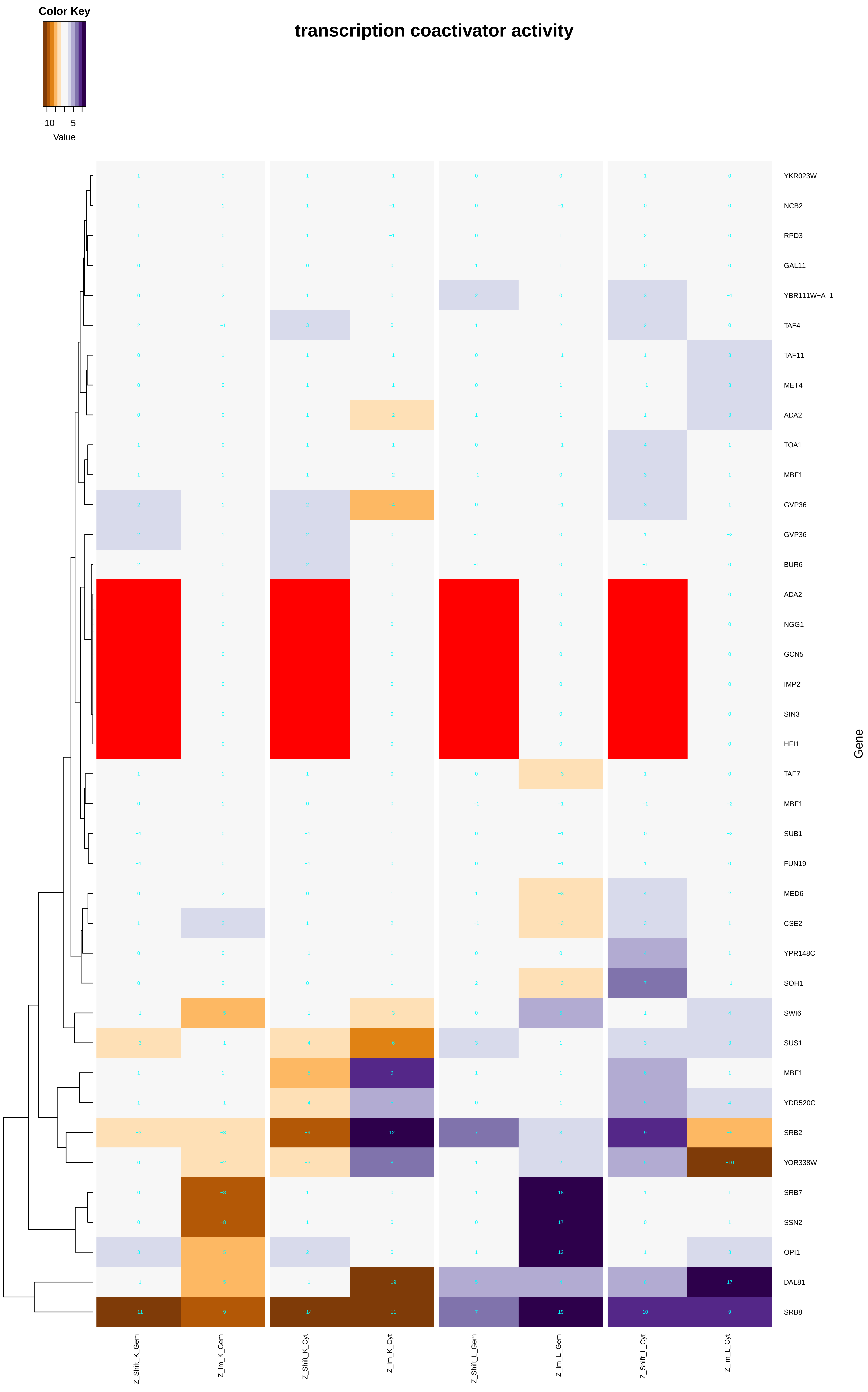

transcription corepressor activity

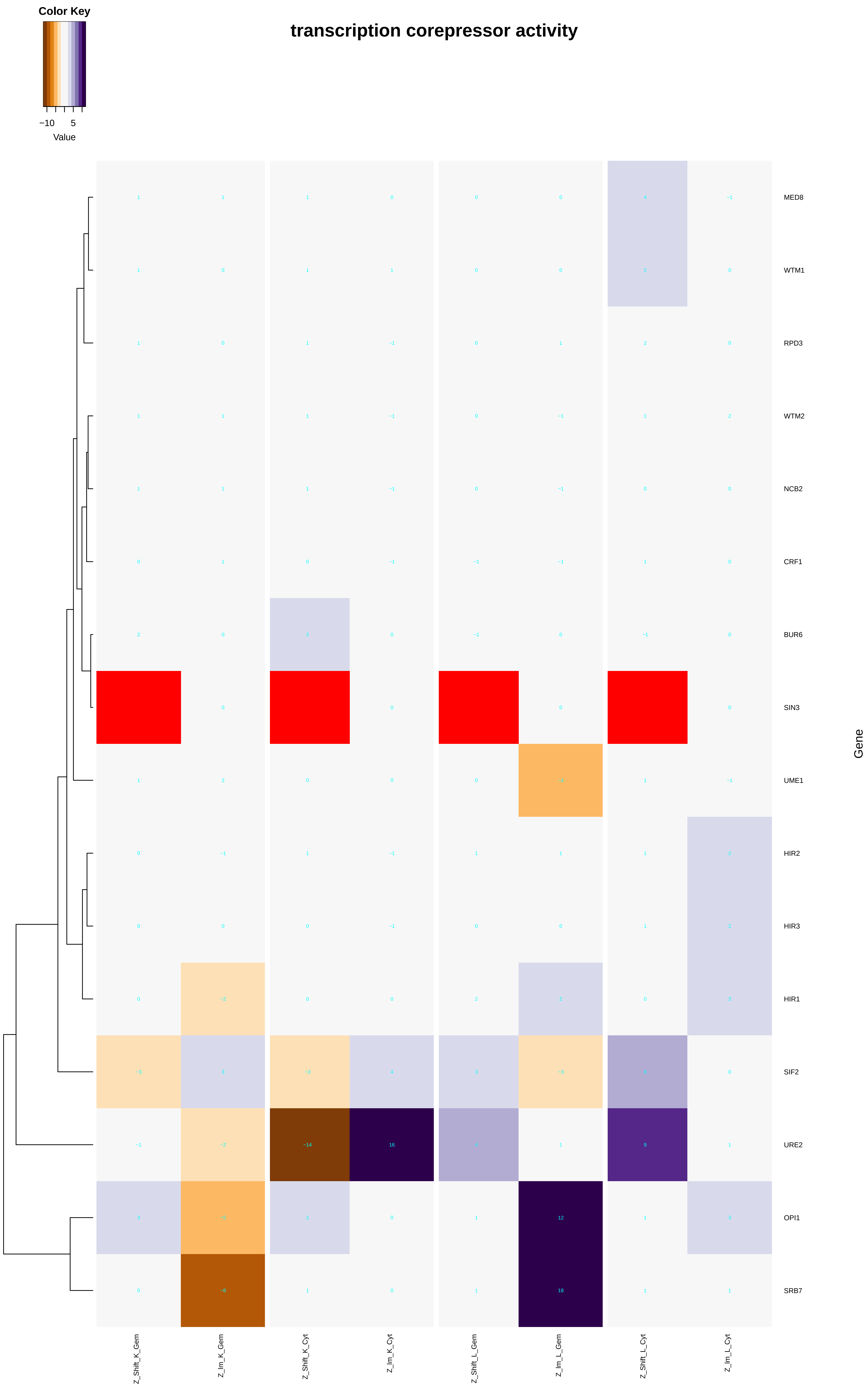

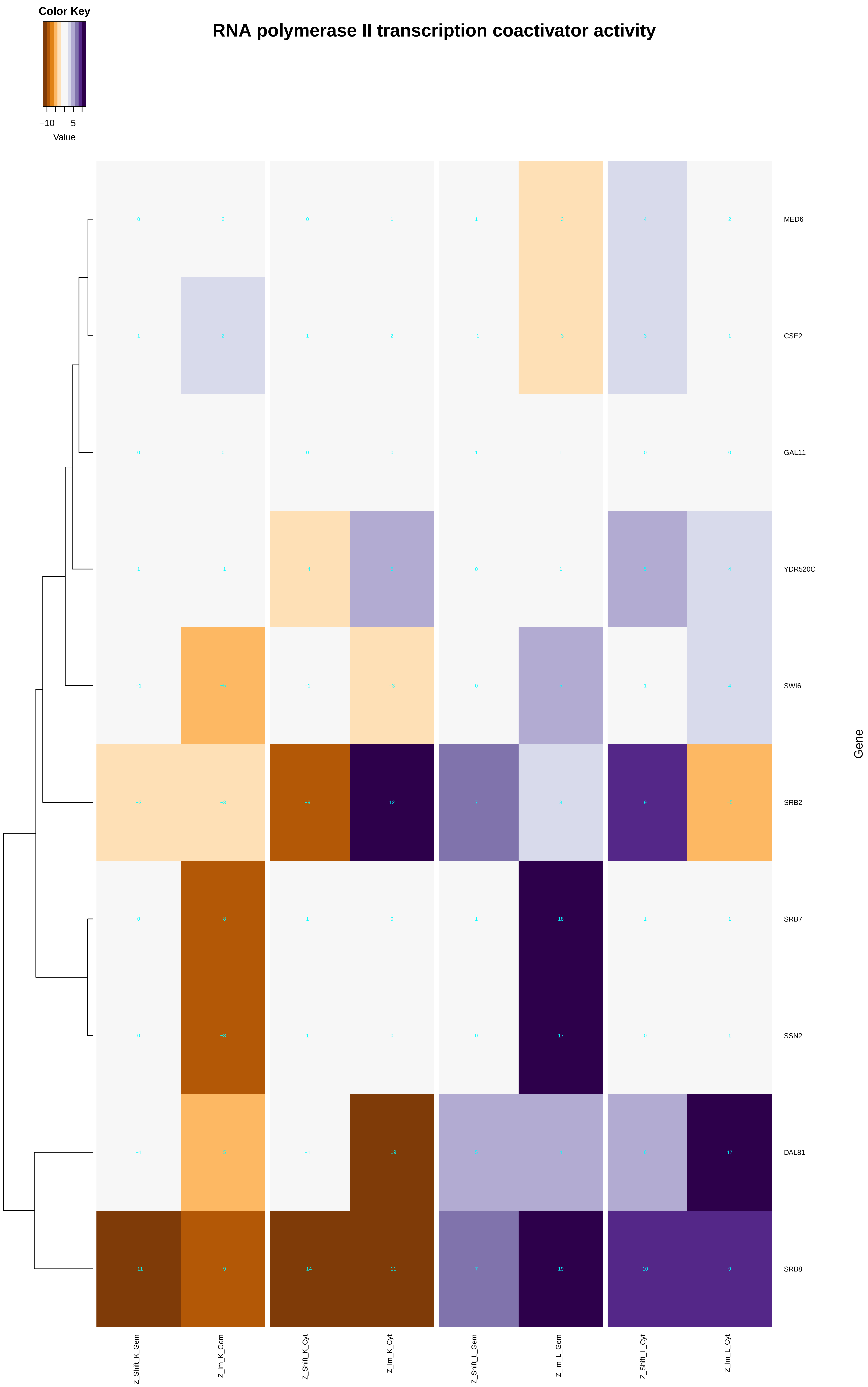

Color Key
−10
5
Value
RNA polymerase II transcription coactivator activity
MED6
CSE2
GAL11
YDR520C
SWI6
SRB2
SRB7
SSN2
DAL81
SRB8
Gene
Z_Shift_K_Gem
Z_Im_K_Gem
Z_Shift_K_Cyt
Z_Im_K_Cyt
Z_Shift_L_Gem
Z_Im_L_Gem
Z_Shift_L_Cyt
Z_Im_L_Cyt

RNA polymerase II transcription corepressor activity

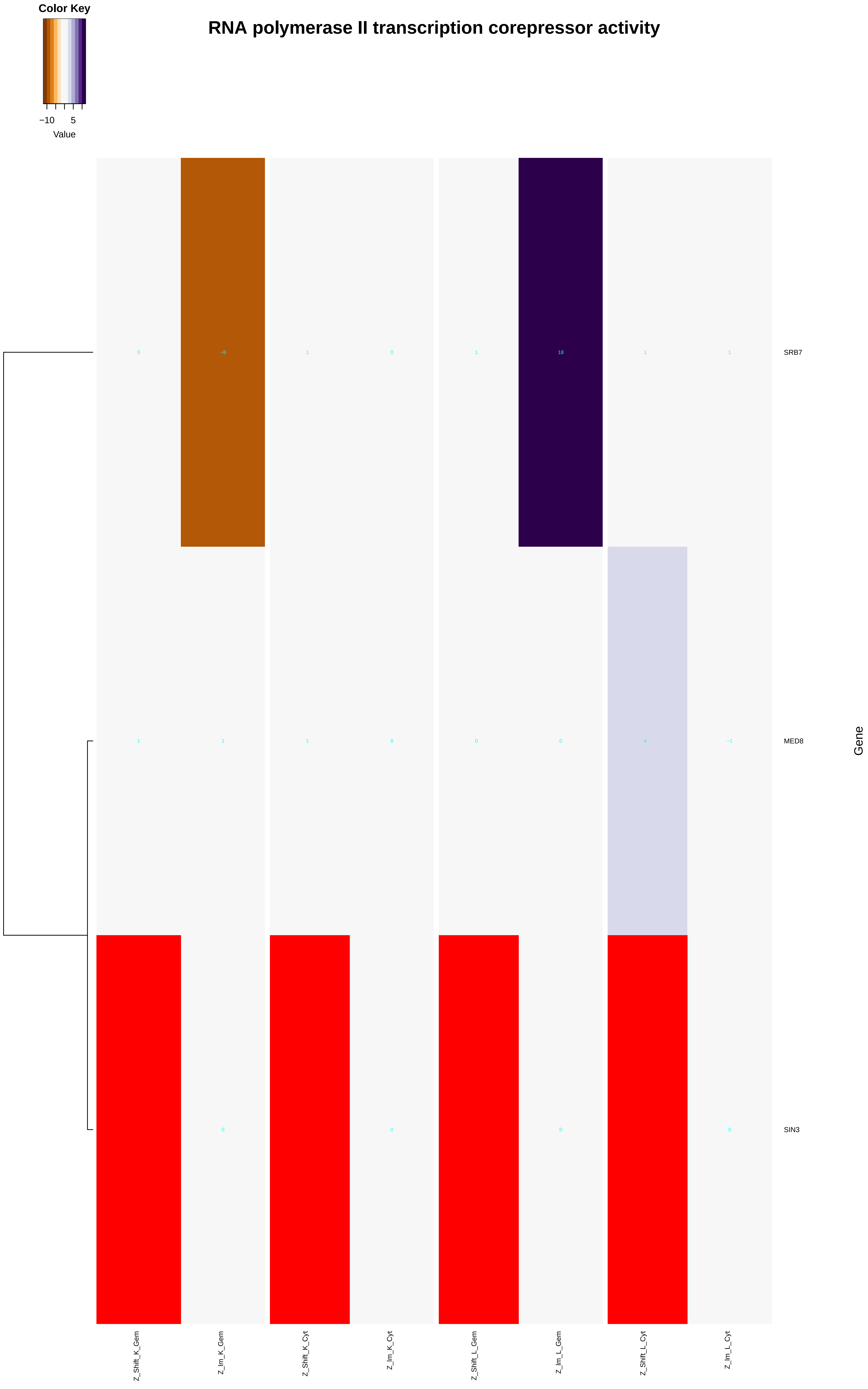

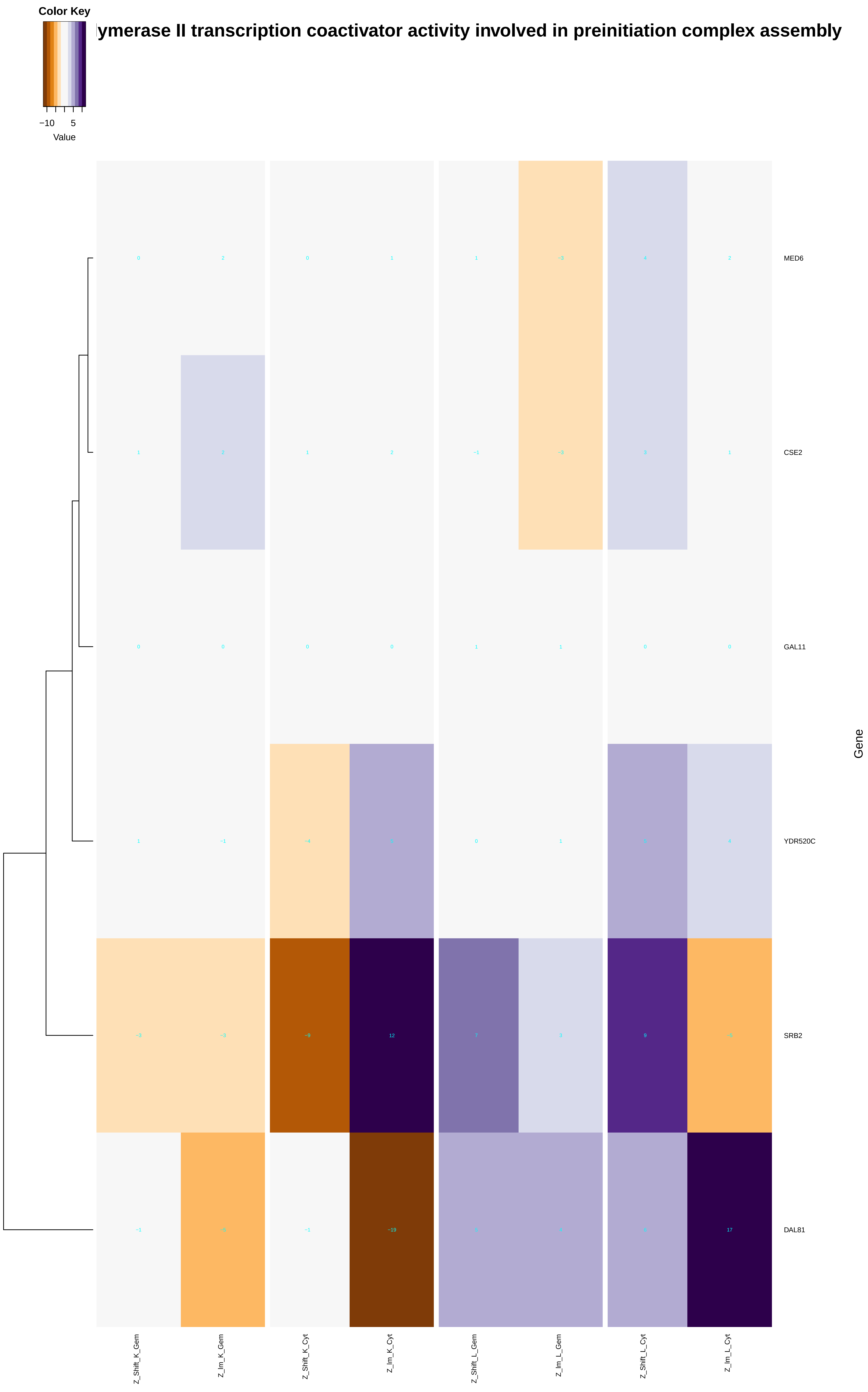

Color Key
−10
5
Value
lymerase II transcription coactivator activity involved in preinitiation complex assembly
MED6
CSE2
GAL11
YDR520C
SRB2
DAL81
Gene
Z_Shift_K_Gem
Z_Im_K_Gem
Z_Shift_K_Cyt
Z_Im_K_Cyt
Z_Shift_L_Gem
Z_Im_L_Gem
Z_Shift_L_Cyt
Z_Im_L_Cyt
